# Comprehensive identification of microbial and metabolomic factors impacting ICC recurrence

**DOI:** 10.3389/fonc.2025.1703182

**Published:** 2026-02-12

**Authors:** Yuan Dang, Shaohua Xu, Jingyun Huang, Xing Peng, Yong Yang, Yingchao Wang, Yanan Yan, Fengle Jiang, Jianmin Wang, Jingfeng Liu

**Affiliations:** 1 Innovation Center for Cancer Research, Clinical Oncology School of Fujian Medical University, Fujian Cancer Hospital, Fuzhou, Fujian, China; 2 Fujian Key Laboratory of Advanced Technology for Cancer Screening and Early Diagnosis, Clinical Oncology School of Fujian Medical University, Fujian Cancer Hospital, Fuzhou, Fujian, China; 3 Department of Hepatopancreatobiliary Surgery, Clinical Oncology School of Fujian Medical University, Fujian Cancer Hospital, Fuzhou, Fujian, China; 4 College of Computer and Data Science, Fuzhou University, Fuzhou, Fujian, China; 5 The United Innovation of Mengchao Hepatobiliary Technology Key Laboratory of Fujian Province, Mengchao Hepatobiliary Hospital of Fujian Medical University, Fuzhou, Fujian, China

**Keywords:** intrahepatic cholangiocarcinoma, metabolome, microbiome, multiomics, progression

## Abstract

**Introduction:**

Intrahepatic cholangiocarcinoma (ICC) originates from intrahepatic bile duct epithelial cells and its global incidence is rising. Surgery remains the primary treatment, but postoperative recurrence rates remain high.

**Methods:**

We analyzed ICC patients' gut microbiota at four stages (preoperative, 7 days postoperative, 1 month postoperative, and during recurrence) using 16S rRNA sequencing and their serum metabolome via LC-MS/MS. Correlations among gut microbiota, metabolome, and clinical indicators were investigated, and candidate microorganisms and metabolites were integrated for multiomics clustering and staging.

**Results:**

This revealed significant increases in Bacteroides, Veillonella, and Enterococcus in ICC patients compared to healthy controls across all stages, suggesting these bacteria as potential markers of ICC progression. Microbial and metabolite changes were observed, with gut microbes influencing ICC development through kynurenic acid, linoleic acid, creatine, cholic acid, L-arginine, and the tumor microenvironment. Multiomics analysis showed that cholangiocarcinoma staging improves patient prognosis, particularly highlighting bile acids' role in type II hepatic phenotypes related to cholesterol metabolism.

**Discussion:**

Our study provides insights into ICC microbiome and metabolome associations with clinical features and survival.

## Background & summary

Intrahepatic cholangiocarcinoma (ICC) is the second most common primary liver malignancy after hepatocellular carcinoma (HCC), accounting for approximately 10% to 20% of all newly diagnosed liver cancer cases. In recent years, the incidence and mortality of ICC have continued to rise. Due to the lack of effective early diagnostic methods, most patients are diagnosed at advanced stage, leading to poor prognosis (1). Although surgical resection is considered the standard treatment for ICC (2), the 5-year survival rate remains only 20% to 35% (3, 4). The high postoperative recurrence rate, ranging from 54% to 71%, is a major contributing factor to the low overall survival rate (5). Therefore, addressing the recurrence of intrahepatic cholangiocarcinoma after surgical resection remains an urgent challenge.

Recent studies have revealed that the intestinal symbiotic ecosystem plays a critical role in pathophysiological processes, nutritional status, immune responses, and disease interactions (6–8). In the field of ICC molecular pathogenesis, the application of next-generation sequencing technology has enabled the identification of specific molecular pathological changes, paving the way for new molecular targeted treatment strategies that may improve patient prognosis (9). Growing evidence suggested that the composition of microbial communities was altered during carcinogenesis or disease progression (10). For instance, studies have characterized the tumor-associated microbiome and elucidated its importance in predicting the prognosis of ICC patients (11).

Furthermore, metabolites may play a crucial role in the occurrence and development of intrahepatic cholangiocarcinoma (ICC). Research has demonstrated that specific changes in serum concentrations of certain metabolites can aid in identifying ICC (12). Additionally, a deeper understanding of linoleic acid pathway disorders and their relationship with liver function changes can provide important therapeutic targets for ICC (13). Abnormalities in these metabolites are not only implicated in tumor cell growth, invasion, and metastasis but also have potential as biomarkers, offering new insights and methods for early diagnosis, disease monitoring, and treatment evaluation of ICC.

With the advancement of high-throughput sequencing technology, the study of multi-omics features has facilitated the identification of molecular subtypes in ICC patients, thereby providing more precise treatment options, including targeted therapy and immunotherapy. For instance, a study (14) analyzed multiple single-cell transcriptomic and auxiliary omics data to identify new molecular subtypes of ICC and validated APOE+C1QB+ tumor-associated macrophages (TAMs) as potential immune therapeutic targets for ICC. Additionally, research on the water extract of Poria cocos (PCD) aimed to investigate whether it improves chronic sleep deprivation (CSD)-induced anxiety behavior by analyzing inflammation factors, metabolic parameters, and gut microbiota. Conjoint analysis revealed a close relationship between these factors (15).

Despite these advances, integrated analyses simultaneously capturing dynamic changes in gut microbiota and systemic metabolism across different treatment stages of ICC remain limited, particularly in the context of postoperative recurrence. To comprehensively investigate whether intestinal microbes and metabolites influence the development of ICC, this study examined the fecal microbiome and serum metabolome. The primary objective was to identify microbial and metabolic features associated with recurrence and to characterize their temporal dynamics across different disease stages. This study was designed to explore intrinsic associations and to provide a systematic, hypothesis-generating framework for future mechanistic and translational investigations.

## Results

### Microbiological analysis of intrahepatic cholangiocarcinoma

To investigate the important marker microorganisms associated with ICC, we conducted 16S rRNA sequencing and microbiological analysis on 117 stool samples from ICC patients and 50 samples from normal individuals. The samples were categorized as follows: 50 from the normal group (N), 20 from the preoperative group (Pre-OP), 36 from the postoperative day 7 group (Post-7D), 38 from the postoperative 1–3 months group (Post-1M), and 23 from the post-recurrence group (Post-R). Samples from the Pre-OP, Post-7D, Post-1M, and Post-R groups were collectively classified as cancer sample groups (non-N).

**Figure 1:**
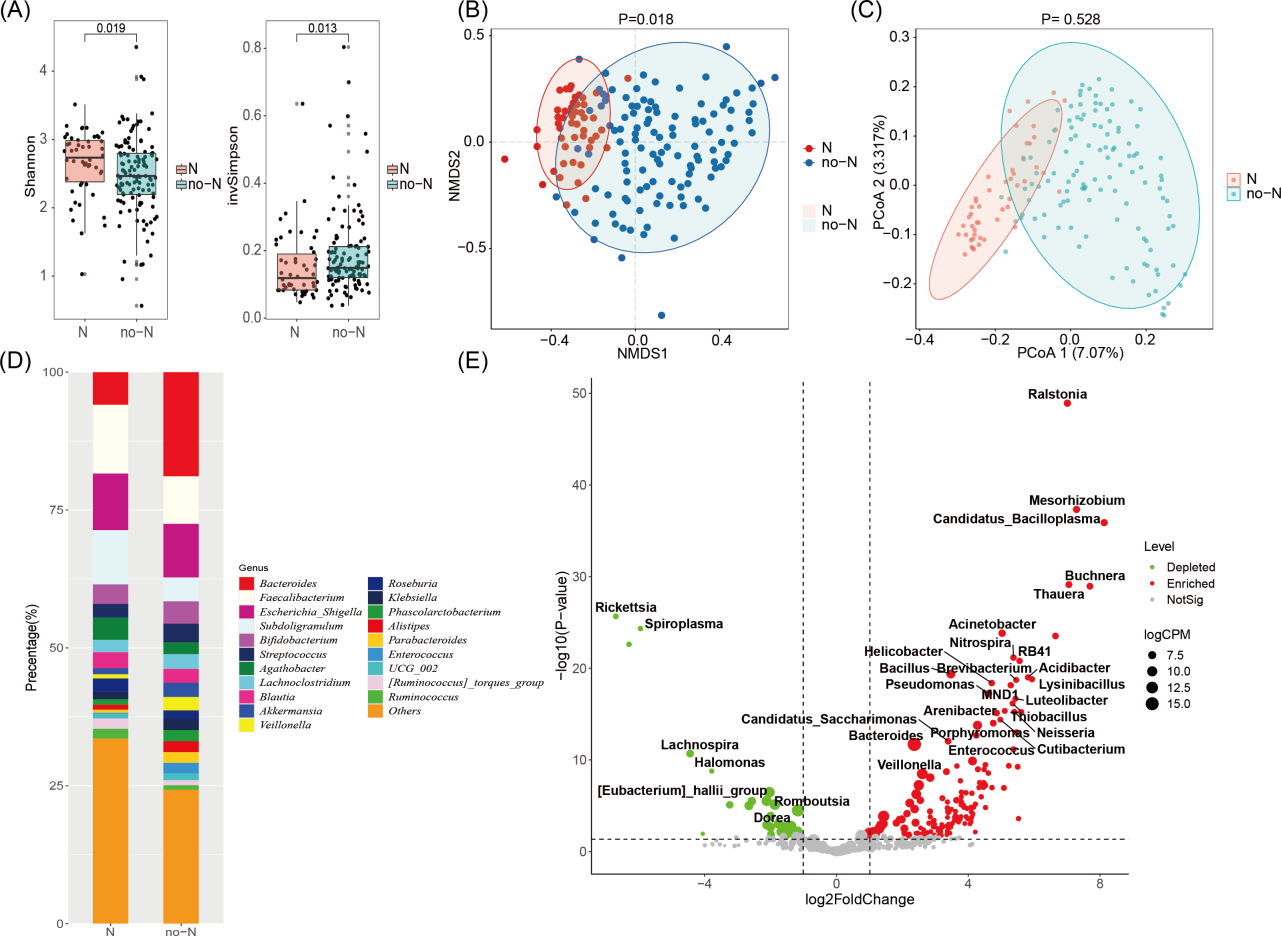
Characterization of the microbiome in non-normal versus normal groups of ICC patients. **(A)** Box plots showing Shannon’s index and Invsimpson’s index (InvSimpson) for the non-normal (no-N) group versus the normal (N) group. **(B)** Nonmetric multidimensional scaling (NMDS) analysis based on the Canberra distance between the no-N and N groups. **(C)** Principal coordinate analysis (PCoA) based on the Canberra distance between the no-N and N groups. **(D)** Stacked histogram depicting the proportions of the top 20 taxa with the highest relative abundance at the genus level in the no-N and N groups. **(E)** Volcano plot of bacterial abundance at the genus level comparing the no-N group to the N group. The cutoff conditions were as follows: |log2-fold change| > 1, p < 0.05. Bacteria that were significantly reduced in the no-N group are shown in green, while those significantly enriched in the no-N group are shown in red.

For the microbiome α diversity analysis of the normal (N) and non-normal (no-N) groups, we used Shannon’s index and invSimpson’s index, which revealed significant differences (p=0.019, p=0.013; **Figure 1A**). For β diversity analysis based on the Canberra distance, NMDS analysis showed significant differences between the no-N and N groups (p=0.018; **Figure 1B**), while PCoA analysis did not show significant differences (p=0.528; **Figure 1C**).

Additionally, we screened the top 20 bacteria based on genus abundance and calculated their proportions in both the no-N and N groups. We found that Faecalibacterium, Bacteroides, Escherichia_Shigella, and Subdoligranulum had the highest proportions and exhibited the largest differences in abundance between the subgroups (**Figure 1D**).

Finally, we performed a difference-in-differences analysis using DESeq2, which revealed significant changes in the abundance of several genera between the no-N and N groups. Specifically, the abundances of Acinetobacter, Bacteroides, Veillonella, and Enterococcus were significantly increased in the no-N group, while the abundances of [Eubacterium]_hallii_group, Dorea, Halomonas, Lachnospira, and Rickettsia were significantly decreased in the no-N group (**Figure 1E**).

Considering the potential changes in the bacterial community across different time periods in the no-N group, we analyzed microbial comparisons between the N group and the preoperative (Pre-OP), postoperative day 7 (Post-7D), postoperative month 1 (Post-1M), and post-recurrence (Post-R) groups.

The alpha diversity analysis based on Shannon’s index and invSimpson’s index showed no significant differences between Pre-OP and N (p=0.29, p=0.21; **Supplementary Figure S1A**); Post-7D and N (p=0.20, p=0.095; **Supplementary Figure S1A**); and Post-1M and N (p=0.051, p=0.069; **Supplementary Figure S1A**). However, there was a significant difference between Post-R and N (p=0.013, p=0.015; **Supplementary Figure S1A**).

The NMDS analysis and PCoA analysis based on the Canberra distance revealed no significant difference between Pre-OP and N (p=0.549; **Supplementary Figure S1B**), but PCoA analysis showed a significant difference (p=0.001; **Supplementary Figure S1C**). For Post-7D and N, NMDS analysis showed no significant difference (p=0.672; **Supplementary Figure S1B**), while PCoA analysis indicated a significant difference (p=0.023; **Supplementary Figure S1C**).

The abundances of Faecalibacterium, Bacteroides, Escherichia_Shigella, and Subdoligranulum were highest in both the N subgroup and other subgroups, with the largest differences detected (**Supplementary Figure S1D**). Finally, variance analysis showed that Bacteroides and Brevibacterium were significantly more abundant in the other subgroups compared to the N subgroup, while [Eubacterium]_hallii_group and Dorea were significantly less abundant in the no-N group compared to the N group.

The analysis revealed that the abundance of Bacteroides was significantly higher in the no-N group compared to the other groups (**Figure 1E**; **Supplementary Figure S1E**).

### Recurrence analysis of intrahepatic cholangiocarcinoma

To investigate the dynamics of microorganisms in ICC across different time periods, we excluded bacteria that appeared in fewer than 10% of the samples and analyzed the 16S sequencing results from 117 fecal samples collected from ICC patients [including the preoperative (Pre-OP), postoperative day 7 (Post-7D), postoperative month 1 (Post-1M), and post-recurrence (Post-R) groups)].

**Figure 2:**
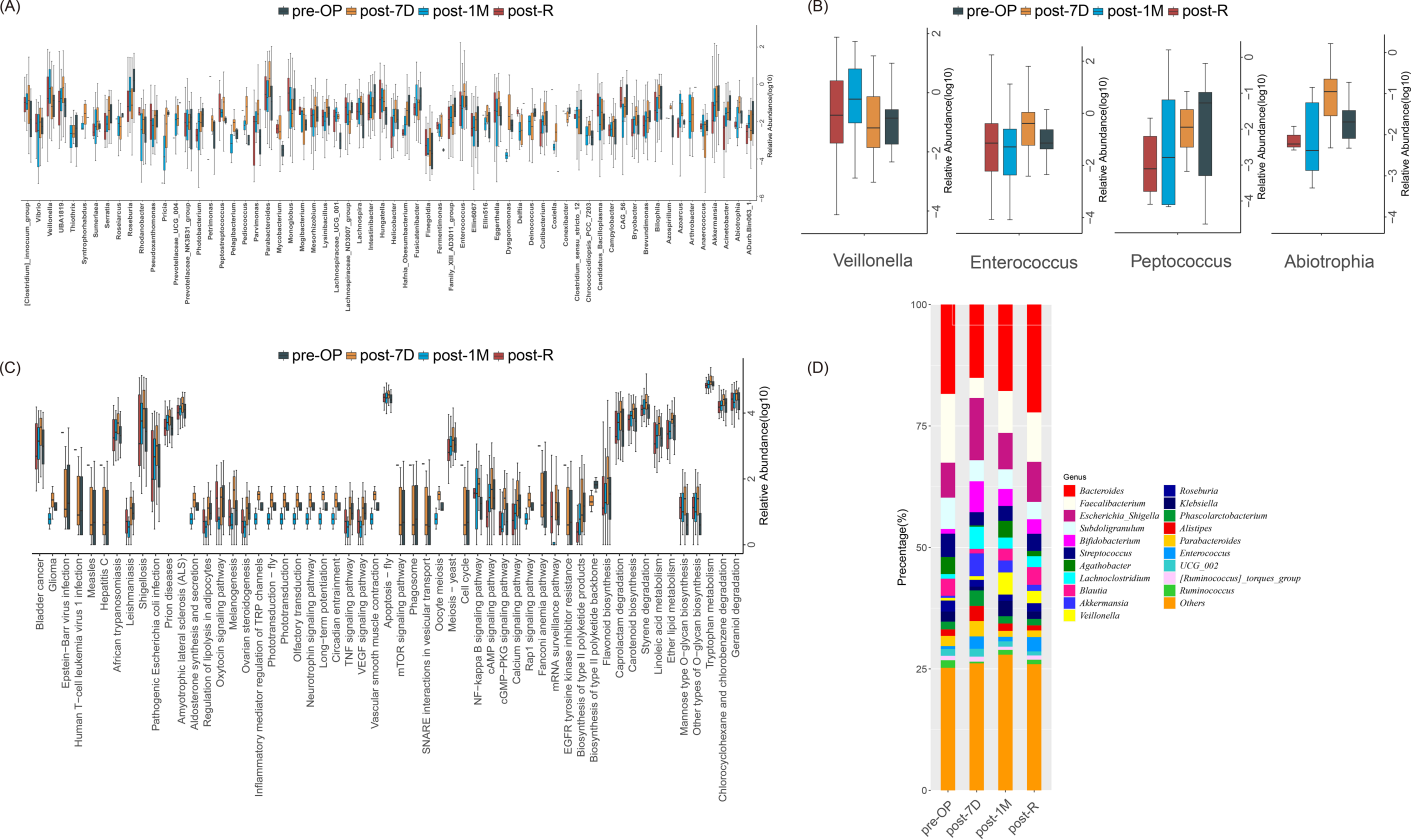
Microbiological analysis of 16S sequencing data from ICC patients at different time points. **(A)** Box plots showing all differentially abundant bacteria identified using the Kruskal-Wallis test (p < 0.05), highlighting bacteria with significant differences in relative abundance among the four groups. **(B)** Box plots illustrating differences in the relative abundances of Veillonella, Enterococcus, Peptococcus, and Abiotrophia across the four time points. **(C)** Box plot of KEGG-based differential pathway analysis, where p < 0.05 indicates statistical significance. **(D)** Stacked histogram depicting the proportions of the top 20 taxa with the highest relative abundance at the genus level over time.

We explored the trends in bacterial abundance in ICC patients during different periods of surgical treatment, including preoperative (Pre-OP), postoperative day 7 (Post-7D), postoperative month 1 (Post-1M), and post-recurrence (Post-R) groups. By analyzing changes in bacterial abundance during these periods, we aimed to gain insights into the effects of surgical treatment on the patients’ microbiota and the dynamics of bacterial populations before and after postoperative recurrence, as well as to identify potential microbial markers. Boxplots showing significant differences in the relative abundances of Veillonella, Enterococcus, Peptococcus, and Abiotrophia are presented in **Figures 2A, B**. Notably, Enterococcus and Abiotrophia exhibited a significant decrease in abundance at Post-1M and a significant increase in abundance post-recurrence.

In addition, we screened the top 20 bacteria based on genus abundance and calculated their proportions across different subgroups. Bacteroides, Veillonella, and Enterococcus were highly represented in all four periods, with significant variations in their abundance proportions (**Figure 2D**). This study further demonstrates that the abundance of Bacteroides could serve as an important marker for ICC.

Finally, based on the KEGG and Greengenes databases, we used PICRUSt to predict the functional characteristics of the microbiome. Using the Kruskal-Wallis test, we identified several functional pathways with significant differences among the four groups, including ether lipid metabolism, linoleic acid metabolism, tryptophan metabolism, mannose-type O-glycan biosynthesis, geraniol degradation, and biosynthesis of type II polyketide backbone (**Figure 2C**). These pathways are closely related to metabolic processes, supporting key life functions such as energy conversion, biomolecule synthesis, and catabolism, and exerting important effects on cellular biological functions and physiological states.

### Validation analysis of microbial community recurrence in intrahepatic cholangiocarcinoma

To more efficiently and accurately study the structure, diversity, and function of the microbial community composition in cholangiocarcinoma patients, we performed metagenomic sequencing on 56 fecal samples collected from these patients. The samples were categorized into four periods: preoperative (Pre-OP, n=11), postoperative day 7 (Post-7D, n=10), postoperative month 1 (Post-1M, n=18), and post-recurrence (Post-R, n=17).

**Figure 3:**
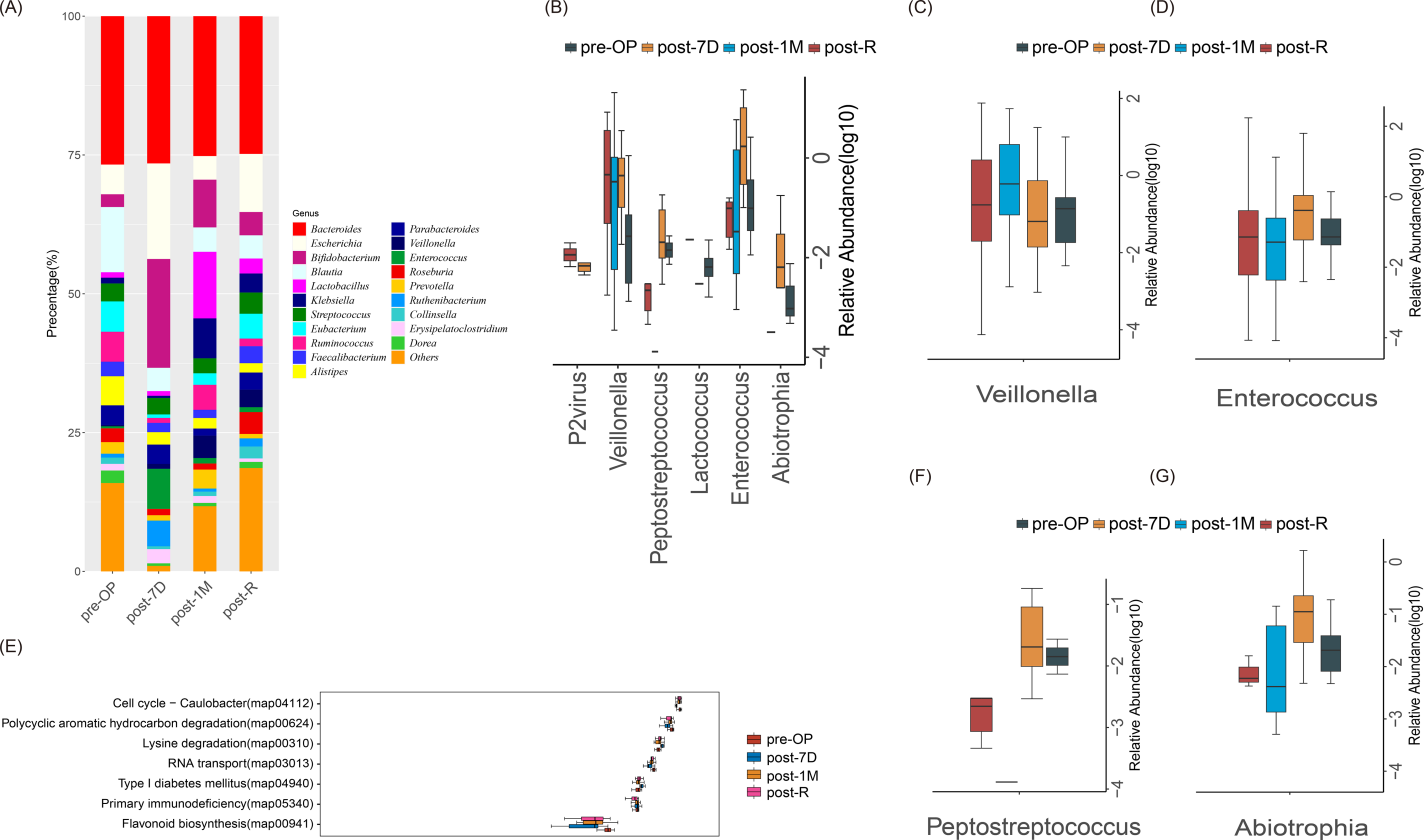
Microbial analysis of ICC metagenomes at different time points. **(A)** Stacked histogram showing the proportions of the top 20 taxa with the highest relative abundance at the genus level across different time points. **(B)** Box plots of all differentially abundant bacteria identified using the Kruskal-Wallis test (p < 0.05), highlighting bacteria with significant differences in relative abundance among the four groups. **(C, D, F, G)** Box plots illustrating differences in the relative abundances of Veillonella, Enterococcus, Peptococcus, and Abiotrophia across the four time points. **(E)** Box plot of KEGG-based differential pathway analysis, where p < 0.05 indicates statistical significance.

We screened the top 20 bacteria based on genus abundance and calculated their proportions across the different subgroups. Bacteroides, Veillonella, and Enterococcus were highly represented in all four periods, exhibiting significant differences (**Figure 3A**). This finding underscores the importance of Bacteroides in ICC biomarker studies and confirms its potential value as a diagnostic and prognostic marker for ICC.

In addition, we investigated the trends in bacterial abundance in cholangiocarcinoma patients across different surgical treatment periods. Bacteria with significant differences in relative abundance at the genus level among the four groups were Abiotrophia, Enterococcus, Lactococcus, Peptostreptococcus, and Veillonella. Our study demonstrated that changes in the abundance of Veillonella, Enterococcus, Peptococcus, and Abiotrophia during different stages of cholangiocarcinoma can serve as important markers for disease diagnosis or monitoring treatment effectiveness. Box plots of the relative abundances of these bacteria are shown in **Figures 3C, D, F, G**. Consistent with previous 16S sequencing results, the abundance of Enterococcus significantly decreased in the postoperative month 1 (Post-1M) group and significantly increased after recurrence, indicating its potential to predict recurrence in ICC patients.

Finally, based on the KEGG and Greengenes databases, we used PICRUSt to predict the functional characteristics of the microbiome. Using the Kruskal-Wallis test, we identified significantly different functional pathways among the four groups, including polycyclic aromatic hydrocarbon degradation, lysine degradation, flavonoid biosynthesis, and others (**Figure 3E**). These pathways primarily influenced metabolic functions, indicating that the biological functions of microbial communities in different periods were closely related to metabolic processes. This study demonstrated that these metabolic processes had important impacts on the function and stability of the ecosystem.

### Metabolite recurrence in intrahepatic cholangiocarcinoma

Metabolic changes at different time points can reflect metabolic regulation during tumor growth, treatment response, and recurrence. To investigate the temporal changes in metabolites in ICC patients and identify early biomarkers for timely adjustment of therapeutic regimens, we conducted QE-based metabolomics analysis on 220 serum samples from a cohort of ICC patients. The samples were categorized into four periods: preoperative (Pre-OP, n=75), postoperative day 7 (Post-7D, n=73), postoperative month 1 (Post-1M, n=47), and post-recurrence (Post-R, n=25). To eliminate uncertainties and biases and to standardize the data within a specific range, we performed centering and standardization preprocessing on the data.

**Figure 4:**
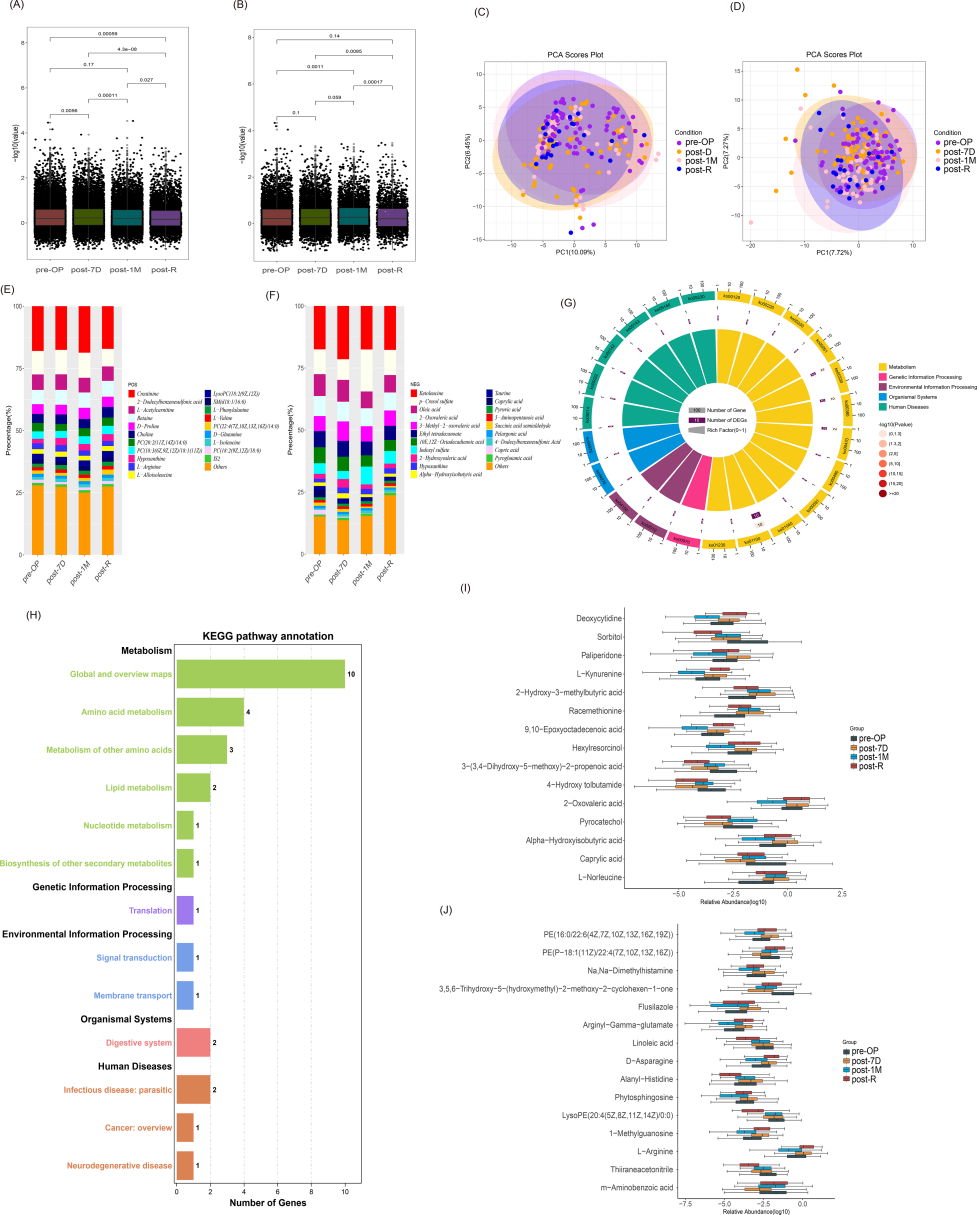
Metabolomic analysis of ICC. **(A)** Box plots showing metabolite abundance differences among the four periods in positive ion mode (POS), analyzed using the Wilcoxon rank sum test. **(B)** Box plots illustrating metabolite abundance differences among the four periods in negative ion mode (NEG), analyzed using the Wilcoxon rank sum test. **(C)** PCA plot for the four periods in positive ion mode (POS). **(D)** PCA plot for the four periods in negative ion mode (NEG). **(E)** Stacked histograms depicting the proportions of the top 20 metabolites with the highest relative abundance across different periods in positive ion mode (POS). **(F)** Stacked histogram showing the proportions of the top 20 metabolites with the highest relative abundance across different periods in negative ion mode (NEG). **(G)** KEGG enrichment analysis with statistical plots for B-level pathway classification. **(H)** Graph of the top 20 significantly enriched pathways from KEGG enrichment analysis, represented as circles. **(I)** Box plots of differentially abundant metabolites in positive ion mode (POS), identified using the Kruskal-Wallis test, highlighting the top 15 metabolites with significant differences in relative abundance among the four groups (p < 0.05). **(J)** Box plots of differentially abundant metabolites in negative ion mode (NEG), identified using the Kruskal-Wallis test, highlighting the top 15 metabolites with significant differences in relative abundance among the four groups (p < 0.05).

Differential analysis of metabolite abundance in different periods revealed significant differences in positive ion mode (POS) between pre-OP and post-1M, post-1M and post-R, and post-7D and post-R (p=0.0011, p=0.00017, p=0.0085; **Figure 4A**). In negative ion mode (NEG), significant differences were observed between pre-OP and post-7D, post-7D and post-1M, pre-OP and post-R, post-1M and post-R, and post-7D and post-R (p=0.0056, p=0.00011, p=0.00059, p=0.027, p=4.3e-08; **Figure 4B**). Additionally, there were significant differences between other periods compared to post-R (p<0.05). PCA analyses showed that the linear relationship between metabolite abundance across the four periods was not strong in either POS or NEG modes (**Figures 4C, D**). Furthermore, we screened the top 20 most abundant metabolites in both POS and NEG modes and calculated their proportions in different periods. In POS mode, creatinine and L-arginine had the highest proportions and exhibited the greatest differences in abundance across subgroups (**Figure 4E**). In NEG mode, hypoxanthine had the highest proportion and showed the greatest difference in abundance across subgroups (**Figure 4F**).

The top 15 metabolites with significant differences in relative abundance among the four groups in positive ion mode (POS) are shown in (**Figure 4I**), and the top 15 metabolites with significant differences in relative abundance among the four groups in negative ion mode (NEG) are shown in (**Figure 4J**). The common differentially abundant metabolites identified in both modes were hypoxanthine, creatinine, D-glutamine, kynurenic acid, N4-acetylcytidine, L-glutamic acid, L-arginine, L-kynurenine, pyroglutamic acid, linoleic acid, and cholic acid. Subsequently, we performed KEGG enrichment analysis on these 11 differentially abundant metabolites, revealing that they primarily influenced metabolic pathways related to amino acid metabolism, lipid metabolism, nucleotide metabolism, and biosynthesis (**Figures 4G, H**).

### Results of the correlation study between microbiological and clinical indicators

**Table 1** presents the clinical characteristics of the ICC samples subjected to 16S sequencing. We measured various clinical markers in ICC patients, including HBV infection status, γ-glutamyl transpeptidase (GGT), alkaline phosphatase (ALP), total cholesterol (TC), monocyte count (MONO), carbohydrate antigen 19-9 (CA19-9), carcinoembryonic antigen (CEA), carbohydrate antigen 125 (CA125), carbohydrate antigen 15-3 (CA15-3), alpha-fetoprotein (AFP), creatine kinase MB isoenzyme (CK-MB), and aspartate aminotransferase (AST). The samples were categorized into four groups based on the timing of measurement: preoperative (Pre-OP, 18%), postoperative day 7 (Post-7D, 31%), postoperative month 1 (Post-1M, 31%), and post-recurrence (Post-R, 20%). Significant differences were observed in creatine kinase MB isoenzyme (CK-MB) and aspartate aminotransferase (AST) levels across different time points (p=0.048 and p=0.049, respectively; **Table 1**).

**Table 1:** Baseline characteristics of samples from different periods.

Characteristics	pre-OP	post-7D	post-1 M	post-R	P
HBV					0.91
HBV-	9 (81.8%)	17 (77.3%)	17 (73.9%)	6 (66.7%)	
HBV+	2 (18.2%)	5 (22.7%)	6 (26.1%)	3 (33.3%)	
GGT					0.548
abnorm	6 (33.3%)	6 (17.6%)	9 (26.5%)	4 (17.4%)	
norm	12 (66.7%)	28 (82.4%)	25 (73.5%)	19 (82.6%)	
ALP					0.846
abnorm	7 (41.2%)	16 (47.1%)	17 (50.0%)	9 (39.1%)	
norm	10 (58.8%)	18 (52.9%)	17 (50.0%)	14 (60.9%)	
TC					0.824
abnorm	3 (15.8%)	9 (26.5%)	8 (21.6%)	4 (17.4%)	
norm	16 (84.2%)	25 (73.5%)	29 (78.4%)	19 (82.6%)	
MONO					0.891
abnorm	4 (21.1%)	6 (17.6%)	5 (13.5%)	4 (18.2%)	
norm	15 (78.9%)	28 (82.4%)	32 (86.5%)	18 (81.8%)	
CA19-9					0.665
abnorm	6 (37.5%)	16 (50.0%)	19 (52.8%)	11 (57.9%)	
norm	10 (62.5%)	16 (50.0%)	17 (47.2%)	8 (42.1%)	
CEA					0.826
abnorm	2 (11.1%)	4 (12.9%)	6 (17.6%)	4 (21.1%)	
norm	16 (88.9%)	27 (87.1%)	28 (82.4%)	15 (78.9%)	
CA125					0.794
abnorm	6 (37.5%)	13 (40.6%)	10 (30.3%)	8 (42.1%)	
norm	10 (62.5%)	19 (59.4%)	23 (69.7%)	11 (57.9%)	
CA15-3					1
abnorm	0 (0.00%)	1 (16.7%)	2 (28.6%)	1 (20.0%)	
norm	2 (100%)	5 (83.3%)	5 (71.4%)	4 (80.0%)	
AFP					0.826
abnorm	16 (94.1%)	26 (86.7%)	31 (91.2%)	17 (85.0%)	
norm	1 (5.88%)	4 (13.3%)	3 (8.82%)	3 (15.0%)	
CMKB					**0.048**
abnorm	1 (5.56%)	13 (38.2%)	6 (17.6%)	5 (21.7%)	
norm	17 (94.4%)	21 (61.8%)	28 (82.4%)	18 (78.3%)	
AST					**0.049**
abnorm	2 (11.1%)	14 (41.2%)	13 (38.2%)	4 (17.4%)	
norm	16 (88.9%)	20 (58.8%)	21 (61.8%)	19 (82.6%)	

Statistically significant P-values are in bold (P < 0.05).

**Figure 5:**
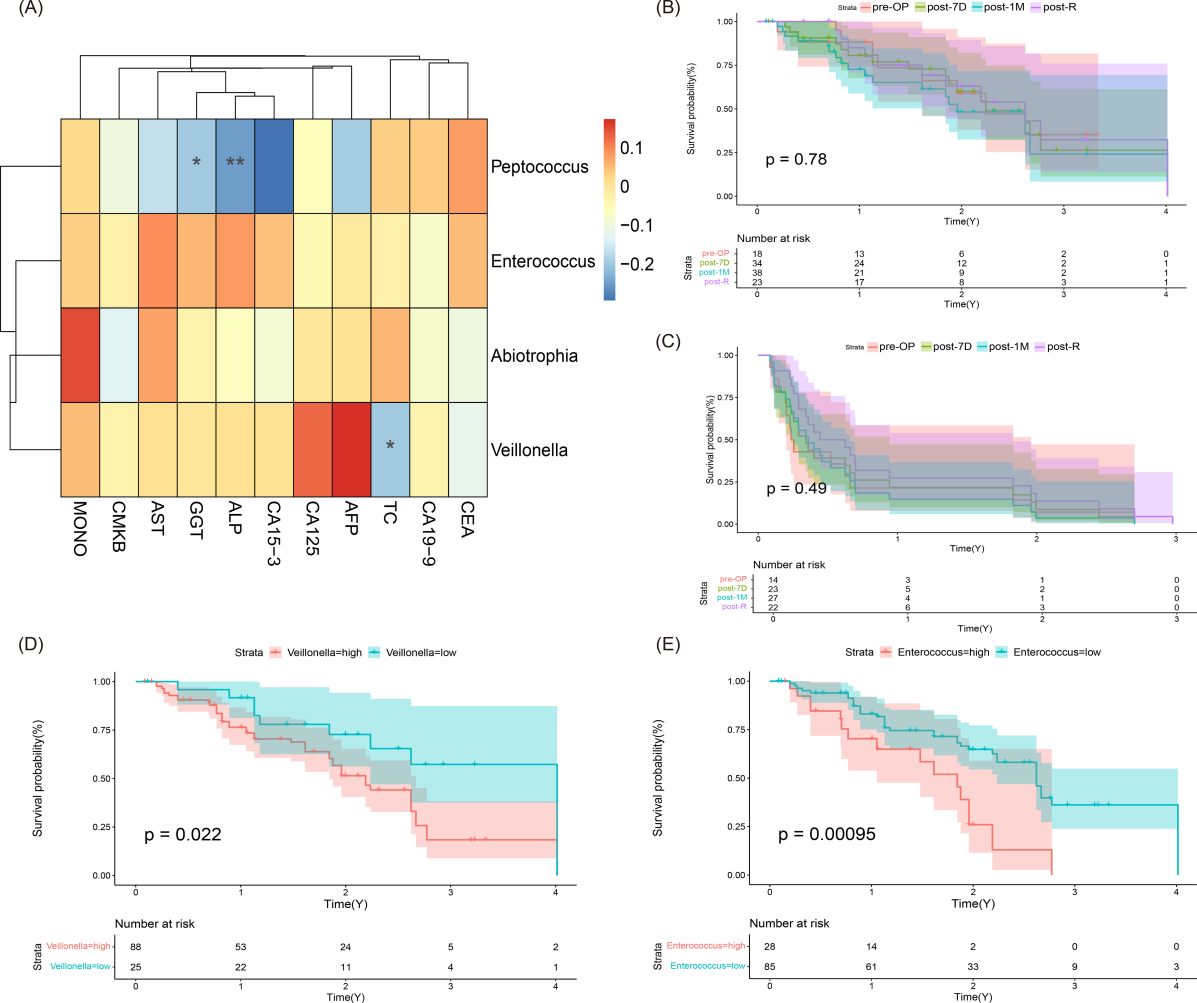
Analysis of microorganisms and clinical correlation in ICC. **(A)** Heatmap illustrating the correlations between significant microorganisms and clinical factors (** indicates p < 0.01, * indicates 0.01 ≤ p < 0.05). **(B)** Overall Survival (OS) analysis graph. **(C)** Recurrence-Free Survival (RFS) analysis graph. **(D)** Survival analysis results for Veillonella at high versus low abundance. **(E)** Survival analysis results for Enterococcus at high versus low abundance.

We integrated significantly differentially abundant bacteria (Veillonella, Enterococcus, Peptococcus, and Abiotrophia) with clinical factors for correlation analysis. Specifically, Peptococcus was significantly associated with GGT, ALP, and CA-153; Veillonella was significantly associated with CA125 and AFP; TC was correlated with Enterococcus, which in turn was significantly associated with AST, ALP, and MONO; Abiotrophia was significantly correlated with MONO (**Figure 5A**). The survival of the four sample periods was examined based on overall survival (OS) and recurrence-free survival (RFS). Kaplan-Meier (K-M) analysis revealed that the differences in survival between periods were not statistically significant (**Figures 5B, C**). Survival analysis based on the abundance of four key marker bacteria indicated that changes in the abundance of Veillonella and Enterococcus could influence the prognosis of cholangiocarcinoma patients (**Figures 5D, E**). Additionally, Cox regression analysis combining clinical factors and bacterial abundance showed that MONO, CA125, and AFP had a significant impact on patient prognosis (p=0.032, p=0.012, p=0.04, **Table 2**). Notably, CA125 emerged as an independent prognostic indicator (**Supplementary Tables S1–S4**).

**Table 2:** Univariate Cox regression analysis for the prediction of OS.

Characteristics	HR	95%CI	P
GGT	1	1-1	0.87
ALP	1	1-1	0.834
TC	0.92	0.72-1.17	0.497
MONO	1.02	1-1.03	**0.032**
CEA	1	0.98-1.02	0.986
CA125	1.01	1-1.01	**0.012**
CMKB	1	0.97-1.03	0.926
AST	1	0.99-1.02	0.777
AFP	1	1-1	**0.04**
HBV	1	1-1	0.874
CA199	1	1-1	0.259
CA153	1.01	0.97-1.04	0.776
Abiotrophia	0.16	0.02-1.13	0.066
Enterococcus	0.9	0.46-1.77	0.756
Peptococcus	1.52	0.7-3.28	0.285
Veillonella	1.8	0.84-3.83	0.129

Statistically significant P-values are in bold (P < 0.05).

### Results of the correlation study between metabolites and clinical indicators

**Table 3** presents the clinical characteristics of the ICC serum samples. We measured various clinical parameters, including HBV infection status, γ-glutamyl transpeptidase (GGT), alkaline phosphatase (ALP), total cholesterol (TC), mononuclear cell count (MONO), carbohydrate antigen 19-9 (CA19-9), carcinoembryonic antigen (CEA), carbohydrate antigen 125 (CA125), carbohydrate antigen 15-3 (CA15-3), and alpha-fetoprotein (AFP) levels. The samples were categorized into four groups based on the timing of collection: pre-operative (pre-OP, 15%), post-operative day 7 (post-7D, 18%), post-operative month 1 (post-1M, 40%), and post-recurrence (post-R, 27%) (**Table 1**).

**Table 3:** Baseline characteristics of samples from different periods.

Characteristics	pre-OP	post-7D	post-1M	post-R	P
HBV					0.819
HBV-	6 (85.7%)	6 (66.7%)	13 (68.4%)	5 (62.5%)	
HBV+	1 (14.3%)	3 (33.3%)	6 (31.6%)	3 (37.5%)	
GGT					0.458
abnorm	3 (30.0%)	2 (16.7%)	9 (33.3%)	3 (15.0%)	
norm	7 (70.0%)	10 (83.3%)	18 (66.7%)	17 (85.0%)	
ALP					0.572
abnorm	6 (60.0%)	7 (58.3%)	16 (59.3%)	8 (40.0%)	
norm	4 (40.0%)	5 (41.7%)	11 (40.7%)	12 (60.0%)	
TC					0.894
abnorm	2 (18.2%)	2 (16.7%)	8 (26.7%)	4 (20.0%)	
norm	9 (81.8%)	10 (83.3%)	22 (73.3%)	16 (80.0%)	
MONO					0.69
abnorm	2 (18.2%)	2 (16.7%)	3 (10.0%)	4 (21.1%)	
norm	9 (81.8%)	10 (83.3%)	27 (90.0%)	15 (78.9%)	
CA19-9					0.785
abnorm	4 (44.4%)	7 (63.6%)	16 (53.3%)	10 (62.5%)	
norm	5 (55.6%)	4 (36.4%)	14 (46.7%)	6 (37.5%)	
CEA					0.493
abnorm	0 (0.00%)	1 (9.09%)	6 (20.7%)	3 (18.8%)	
norm	10 (100%)	10 (90.9%)	23 (79.3%)	13 (81.2%)	
CA125					0.961
abnorm	3 (33.3%)	3 (25.0%)	9 (33.3%)	6 (37.5%)	
norm	6 (66.7%)	9 (75.0%)	18 (66.7%)	10 (62.5%)	
CA15-3					0.824
abnorm	0 (0.00%)	0 (0.00%)	2 (33.3%)	1 (20.0%)	
norm	2 (100%)	4 (100%)	4 (66.7%)	4 (80.0%)	
AFP					0.654
abnorm	9 (100%)	9 (100%)	24 (88.9%)	15 (88.2%)	
norm	0 (0.00%)	0 (0.00%)	3 (11.1%)	2 (11.8%)	

**Figure 6:**
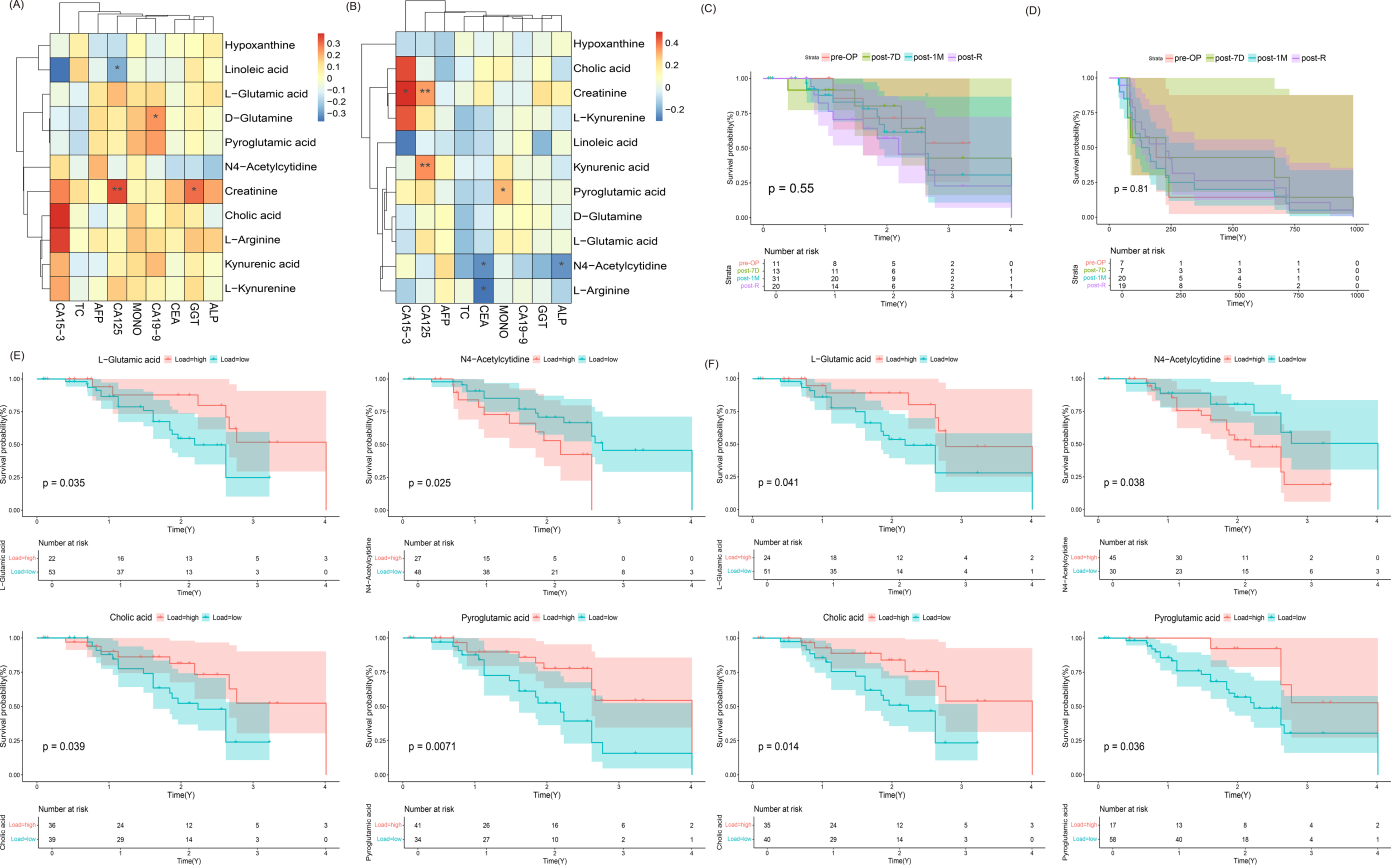
Metabolomic and clinical correlation analysis of cholangiocarcinoma. **(A)** Heatmap illustrating the correlation between key microorganisms and clinical factors in the POS model (** denotes p < 0.01, * denotes 0.01 ≤ p < 0.05). **(B)** Heatmap illustrating the correlation between key microorganisms and clinical factors in the NEG model (** denotes p < 0.01, * denotes 0.01 ≤ p < 0.05). **(C)** Overall Survival (OS) analysis graph. **(D)** Recurrence-Free Survival (RFS) analysis plot. **(E)** Survival analysis results for L-glutamic acid, N4-acetylcytidine, cholic acid, and pyroglutamic acid at high and low abundances in the POS model. **(F)** Survival analysis results for L-glutamic acid, N4-acetylcytidine, cholic acid, and pyroglutamic acid at high and low abundances in the NEG model.

We identified 11 key metabolites (hypoxanthine, creatine, D-glutamine, kynurenic acid, N4-acetylcytidine, L-glutamic acid, L-arginine, L-kynurenine, pyroglutamic acid, linoleic acid, and cholic acid) that were significantly correlated with clinical factors. In the POS model (**Figure 6A**), linoleic acid was significantly correlated with CA125, cholic acid with CA15-3, D-glutamine with CA19-9, and creatine with both GGT and CA125. In the NEG model (**Figure 6B**), creatine was significantly correlated with CA15–3 and CA125, kynurenic acid with CA125, cholic acid with CA15-3, pyroglutamic acid with MONO, N4-acetylcytidine with CEA and ALP, and L-arginine with CEA.

Survival analysis based on overall survival (OS) and recurrence-free survival (RFS) across four sample periods revealed no significant differences between periods (**Figures 6C, D**). Further survival analysis of the 11 differentially abundant metabolites in the POS and NEG models indicated that L-glutamic acid, N4-acetylcytidine, cholic acid, and pyroglutamic acid could influence patient prognosis in ICC (**Figures 6E, F**).

Additionally, Cox regression analysis combining clinical factors and metabolite data showed that CA19-9, CA15-3, and linoleic acid had a significant impact on cholangiocarcinoma prognosis (p=0.019, p=0.011, p=0.01, **Table 4**). Notably, linoleic acid emerged as an independent prognostic indicator (**Table 5**).

**Table 4:** Univariate Cox regression analysis for the prediction of OS.

Characteristics	HR	95%CI	P
GGT(norm vs abnorm)	1.07	0.46-2.46	0.878
ALP(norm vs abnorm)	0.73	0.33-1.6	0.426
TC(norm vs abnorm)	1.16	0.47-2.89	0.75
MONO(norm vs abnorm)	0.28	0.04-2.1	0.214
CEA(norm vs abnorm)	0.38	0.09-1.61	0.187
CA125(norm vs abnorm)	0.93	0.37-2.34	0.879
AFP(norm vs abnorm)	1.27	0.17-9.61	0.814
HBV(HBV- vs HBV+)	0.6	0.19-1.9	0.386
CA199(norm vs abnorm)	2.93	1.2-7.19	**0.019**
CA153(norm vs abnorm)	24.88	2.09-296.49	**0.011**
Hypoxanthine	1	1-1	0.943
Creatinine	1	1-1	0.299
D-Glutamine	1	1-1	0.343
Kynurenic acid	1	1-1	0.643
N4-Acetylcytidine	1	1-1	0.091
L-Glutamic acid	1	1-1	0.649
L-Arginine	1	1-1	0.138
L-Kynurenine	1	1-1	0.639
Pyroglutamic acid	1	1-1	0.13
Linoleic acid	1	1-1	**0.01**
Cholic acid	1	1-1	0.803

Statistically significant P-values are in bold (P < 0.05).

**Table 5:** Multivariate Cox regression analysis for survival prediction of OS patients.

Characteristics	HR	95%CI	P
CA19-9	0.049	0.001-2.22	0.12
CA15-3	6.2	0.5-76.3	0.16
Linoleic acid	1	1-1	**0.043**

Statistically significant P-values are in bold (P < 0.05).

### Results of the correlation study between microorganisms and metabolites

**Figure 7:**
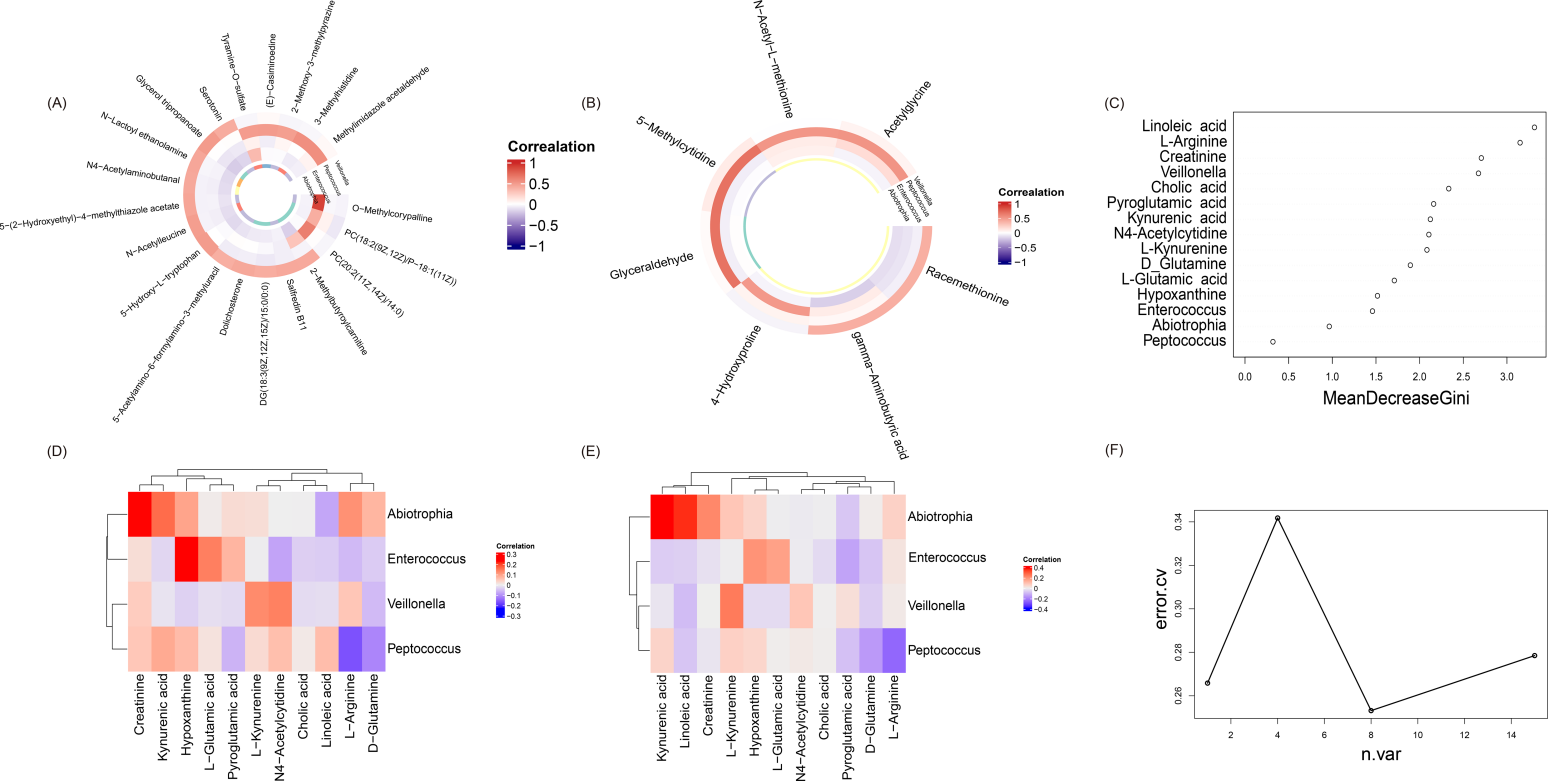
Analysis of microbial and metabolomic correlations in cholangiocarcinoma. **(A)** Metabolites significantly associated with key microorganisms in the POS model. **(B)** Metabolites significantly associated with key microorganisms in the NEG model. **(C)** Scatterplot of significant features, where mean decrease Gini values indicate the relative importance of each variable in predicting recurrence; higher values signify greater influence. **(D)** Heatmap illustrating the correlation between key microorganisms and metabolites in the POS model (** denotes p < 0.01, * denotes 0.01 ≤ p < 0.05). **(E)** Heatmap illustrating the correlation between key microorganisms and metabolites in the NEG model (** denotes p < 0.01, * denotes 0.01 ≤ p < 0.05). **(F)** Line plot showing the 5-fold cross-validation mean error rate as the number of features increases, with the lowest point (minimum loss rate) occurring at 8 features.

Further investigation into the interactions between key microorganisms and metabolites in cholangiocarcinoma revealed significant correlations between four differential bacteria—Veillonella, Enterococcus, Peptococcus, and Abiotrophia—and the levels of 20 metabolites in the POS model (**Figure 7A**). In the NEG model, these bacteria were significantly correlated with the levels of 7 metabolites (**Figure 7B**).

In addition, 11 key metabolites identified in previous experiments were correlated with specific microorganisms. In the POS mode (**Figure 7D**), Abiotrophia exhibited a significant positive correlation with creatine, Enterococcus showed a significant positive correlation with kynurenic acid, and Peptococcus demonstrated a significant negative correlation with L-arginine. In the NEG mode (**Figure 7E**), Abiotrophia was significantly positively correlated with kynurenic acid, linoleic acid, and creatine, while Veillonella was significantly positively correlated with L-kynurenic acid, and Peptococcus remained significantly negatively correlated with L-arginine.

In addition, we constructed a random forest classifier using key metabolites and microorganisms to predict recurrence, and performed 5-fold cross-validation. The scatterplot (**Figure 7C**) illustrates the ranking of important features influencing recurrence prediction. Notably, the model achieved an accuracy of 74.68% when utilizing eight features (**Figure 7F**). These features—linoleic acid, L-arginine, creatinine, Veillonella, cholic acid, pyroglutamic acid, kynurenic acid, and N4-acetylcytidine—demonstrated high predictive accuracy.

### Multiomics subtype analysis was performed by combining the microbiome with the metabolome

**Figure 8:**
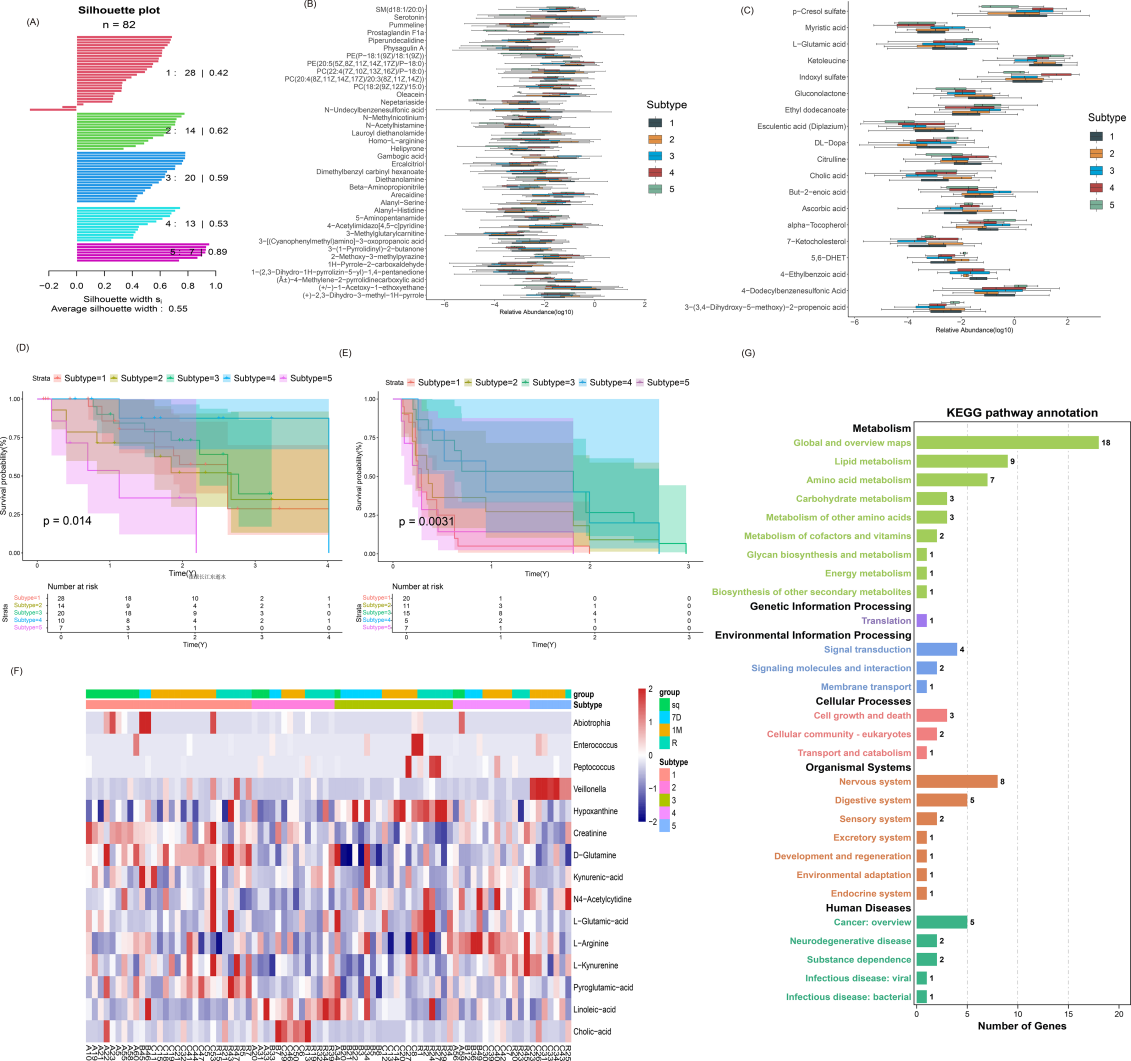
Multihomology analysis. **(A)** Contour plots (silhouette plots) from five replicate clustering experiments. **(B, C)** Box plots of differentially abundant metabolites in POS and NEG modes for the five ICC liver types (p<0.05). **(D)** Overall survival (OS) analysis for the five ICC liver types. **(E)** Recurrence-free survival (RFS) analysis for the five ICC liver types. **(F)** Heatmap showing the distribution of the five ICC liver types. **(G)** KEGG enrichment analysis of key metabolites in the five ICC liver types.

We screened samples from ICC patients using both 16S rRNA sequencing of fecal samples and metabolomic profiling of serum samples. The samples were divided into four groups: pre-OP (n=15), post-7D (n=14), post-1M (n=32), and post-R (n=21), totaling 82 samples. We combined marker microorganisms and key metabolites (averaged from POS and NEG modes) identified in previous experiments for consistent clustering. The average silhouette width from five cross-repeated experiments was 0.55 (**Figure 8A**). Ultimately, we classified these ICC samples into five subtypes.

Metabolite differences were analyzed across the five liver types, revealing 38 significantly different metabolites in the POS mode and 19 in the NEG mode (**Figures 8B, C**). Notably, cholic acid again played a significant role in ICC. Kaplan–Meier survival analysis for the five liver types showed significant differences in overall survival (OS), with the best prognosis for liver type 4 and the worst for liver type 5 (p=0.014, **Figure 8D**). Additionally, recurrence-free survival (RFS) analysis revealed significant differences, with the best prognosis for liver type 3 and the worst for liver type 1 (p=0.0031, **Figure 8E**).

The heatmap distribution of marker microorganisms, metabolites, and subtypes indicated that Veillonella was mainly enriched in class 3 hepatotypes, Enterococcus in class 5 hepatotypes, and cholic acid in class 2 hepatotypes (**Figure 8F**). KEGG enrichment analysis of the differentially abundant metabolites from the five liver types showed that these metabolites were primarily involved in pathways affecting biological processes (**Figure 8G**).

## Discussions

ICC is a malignant tumor originating from intrahepatic biliary epithelial cells, and its pathogenesis remains incompletely understood. However, growing evidence suggests that the microbiome and metabolome play crucial roles in the onset and progression of ICC (16, 17). During ICC development, changes in microbial abundance within intrahepatic bile duct tissues may be closely associated with key pathogenic mechanisms such as chronic inflammation, liver injury, and immune regulation.

In our study, we analyzed fecal samples from ICC patients to explore differences in the microbiota between ICC patients and the normal population. Using 16S rRNA sequencing, we compared the microbial composition of tumor samples (pre-OP, post-7D, post-1M, and post-R groups) with that of the normal group (N). Consistent with previous studies, we found significant differences in Bacteroides abundance between tumor and normal fecal samples. Furthermore, by comparing ICC samples with normal samples at different time points, we identified Bacteroides as a potential biomarker for cholangiocarcinoma. Importantly, although overall alpha diversity differences between most perioperative subgroups and the normal group were modest (**Supplementary Figure S1A**), beta diversity analyses revealed stage-dependent compositional shifts, indicating that microbial community structure rather than overall richness was altered during disease progression (**Supplementary Figures S1B, C**).

By further analyzing changes in the microbiota over time, we observed significant fluctuations in the abundances of Veillonella, Enterococcus, Peptococcus, and Abiotrophia at various stages. Notably, Enterococcus abundance decreased significantly in the post-1M group but increased markedly in the post-R group, suggesting its potential as a biomarker for predicting ICC recurrence. These temporal trends were consistently observed across both the main analysis and subgroup comparisons (**Figures 2A, B**; **Supplementary Figure S1E**), supporting the robustness of Enterococcus-associated recurrence signals rather than isolated time-point effects. As a gram-positive, partially anaerobic coccobacillus, Enterococcus is commonly associated with conditions such as urinary tract infections, bacteremia, and infective endocarditis. Although it is relatively rare in intra-abdominal infections and meningitis, Enterococcus may indirectly contribute to ICC recurrence by triggering inflammatory responses.

To gain insights into the specific genetic composition and functional potential of microorganisms, we employed metagenomic sequencing to characterize the microbiomes of ICC patients at different disease stages. The results indicated that Enterococcus may play a key role in ICC recurrence, with fluctuations in its abundance potentially reflecting changes in the tumor microenvironment and impacting ICC progression. Notably, metagenomic results validated the recurrence-associated patterns identified by 16S sequencing, demonstrating consistent genus-level changes across independent sequencing platforms (**Figures 3A–D**), thereby strengthening the reliability of these findings. Therefore, changes in Enterococcus abundance are expected to serve as an important indicator for predicting ICC recurrence. Additionally, variations in Bacteroides abundance are closely associated with ICC onset and progression, likely reflecting an imbalanced gut microbiome and consequently influencing the disease process.

Through comprehensive 16S rRNA sequencing and metagenomic sequencing, we identified key microbial markers closely associated with different stages of intrahepatic cholangiocarcinoma (ICC), including Veillonella, Enterococcus, Peptococcus, and Abiotrophia. These microorganisms exhibited significant changes during ICC development, reflecting dynamic alterations in the tumor microenvironment and potentially influencing metabolic processes. These findings provide new insights and potential biomarkers for the prevention, diagnosis, and treatment of ICC.

Regarding the research methodology, in addition to analyzing microbiome data, we further investigated the mechanisms by which metabolites influence tumor progression. During different stages of ICC development, the composition and abundance of metabolites, including proteins, lipids, and carbohydrates, may undergo significant changes. These changes are potentially linked to tumor growth, metabolic activities, and tumor-related inflammatory and immune responses. Therefore, we analyzed metabolite profiles in ICC serum samples at various time points. Although principal component analysis did not show strong linear separation among all stages (**Figures 4C, D**), differential abundance analyses identified multiple metabolites with consistent and stage-specific changes, highlighting dynamic metabolic remodeling rather than static group differences. The results showed that 11 metabolites, including kynurenic acid, linoleic acid, creatine, cholic acid, and L-arginine, exhibited significant differences across all four periods under both positive (POS) and negative (NEG) ion modes. These metabolites were primarily involved in amino acid metabolism, lipid metabolism, nucleotide metabolism, and biosynthesis.

Previous studies have conducted two-sample Mendelian randomization (MR) analyses and demonstrated that Veillonella is associated with an increased risk of ICC (18). By investigating the correlations between microorganisms, metabolites, and key clinical factors, we revealed that CA125 can serve as an independent prognostic indicator for ICC. Similarly, changes in the abundance of Veillonella and Enterococcus were shown to influence patient prognosis. Other researchers have found that abnormalities in linoleic acid metabolism may promote the transition from intrahepatic bile duct stones to ICC (13).

Our integrated clinical and metabolomic study further confirmed that linoleic acid concentration can be used as an independent prognostic indicator and is strongly correlated with the tumor marker CA125. Additionally, changes in the levels of four metabolites—L-glutamic acid, N4-acetylcytidine, cholic acid, and pyroglutamic acid—also influence ICC prognosis. These associations were consistently supported by both survival analyses and Cox regression models (**Figures 6E, F**; **Tables 4**, **5**), indicating that metabolite-based prognostic signals were not dependent on a single analytical approach. Notably, there was a strong association between creatine and GGT with CA125. Cholic acid was significantly positively correlated with CA15-3, while L-arginine levels were significantly correlated with carcinoembryonic antigen (CEA).

In addition, our studies in microbiology and metabolomics revealed that increased levels of kynurenic acid were associated with higher abundance of Enterococcus and Veillonella, while elevated L-arginine levels may reduce the abundance of Peptococcus. Furthermore, increases in kynurenic acid, linoleic acid, and creatinine may promote the growth of Abiotrophia. These microbe–metabolite associations were reproducibly observed across both POS and NEG ion modes (**Figures 7A–E**), suggesting stable interaction patterns rather than mode-specific artifacts. These interactions between microorganisms and metabolites play a crucial role in ICC initiation, progression, and therapeutic response. Our findings provide new insights and potential biomarkers for the prevention, diagnosis, and treatment of ICC.

By integrating microbiome and metabolome data, cluster analysis identified distinct sample groups with similar microbial and metabolic profiles. This approach can help define disease subtypes and guide therapeutic strategies for intrahepatic cholangiocarcinoma (ICC). Using previously validated key microbial and metabolite markers, we classified ICC samples into five subtypes and observed significant survival differences between them, indicating practical implications for prognosis. Although the average silhouette width indicated moderate clustering strength (**Figure 8A**), the identified subtypes demonstrated clear and biologically interpretable differences in metabolite composition and survival outcomes, supporting their clinical relevance.

Type 2 hepatotypes exhibited enrichment in bile acids, likely linked to cholesterol metabolism and bile acid synthesis pathways, which may influence liver function and ICC pathogenesis. Type 3 hepatotypes showed higher levels of Veillonella, suggesting an impact on metabolic status or immune response. Type 4 hepatotypes were enriched in Enterococcus, indicating potential associations with inflammatory or infection-related processes, as Enterococcus is commonly associated with various infectious conditions.

These results suggest that changes in microbial composition and metabolite levels characterize different ICC subtypes and may serve as biomarkers for diagnosis, prognosis, and treatment selection. The findings provide new insights into ICC heterogeneity and support the development of personalized therapeutic strategies. Additionally, our study highlights the importance of glutamate and bile acids in ICC. Metabolic pathway enrichment analysis revealed that subtype-specific metabolic differences are closely related to pathogenesis and disease progression.

It is important to acknowledge several limitations of this study when interpreting the observed associations and their potential biological implications. First, stool microbiome and serum metabolome samples were not fully paired at the individual patient level, which limits the strength of integrated microbetedns.ficda correlation analyses. As a result, the relationships identified here should be interpreted as population-level associations rather than direct individual-level interactions, and the present study should be considered hypothesis-generating. Second, ICC patients underwent different clinical interventions across disease stages, including surgical treatment and perioperative management, which may introduce treatment-related confounding effects on both microbial composition and metabolic profiles. Although the longitudinal sampling design partially mitigates this issue by capturing within-disease temporal changes, residual clinical heterogeneity cannot be completely excluded. Third, while healthy fecal samples were included to establish baseline microbial differences, serum metabolomic data from healthy individuals were not available. Therefore, the serum metabolic alterations observed in this study primarily reflect endogenous dynamic changes associated with treatment and recurrence rather than absolute cancer-specific metabolic signatures. Finally, this study was conducted in a single-center cohort with a moderate sample size, particularly in the recurrence subgroup, which may limit statistical power and generalizability. External validation in larger, independent, and preferably multi-center cohorts will be necessary to confirm the robustness and clinical applicability of the identified microbial and metabolic markers.

Taken together, although this study identifies reproducible associations between gut microbiota, serum metabolites, and ICC recurrence, these findings do not establish causal relationships. Future studies incorporating fully paired multi-omics data, healthy serum controls, multi-center validation, and mechanistic experiments will be essential to clarify causal roles and facilitate clinical translation.

## Conclusions

Our analyses provide novel insights into the microbiome and metabolome of intrahepatic cholangiocarcinoma (ICC), revealing associations between specific microbiota, metabolites, and clinical features. Bacteroides, Veillonella, and Enterococcus strains significantly influence ICC, with changes in their abundance serving as potential diagnostic indicators. Specifically, altered levels of Veillonella and Enterococcus predict disease recurrence and are associated with favorable prognosis during four therapeutic phases (pre-OP, post-7D, post-1M, and post-R). Gut microbiota and their metabolites collectively impact ICC progression by modulating tumor microenvironment through compounds like creatine, linoleic acid, bile acids, and L-arginine. Multi-omics analysis shows that the type 2 liver phenotype is influenced by bile acids linked to cholesterol metabolism and bile acid synthesis pathways. These findings highlight the importance of considering ICC heterogeneity and suggest the potential value of intestinal microorganisms and metabolites in diagnosis, prognosis assessment, and therapeutic strategies for ICC.

## Materials and methods

### Sample collection

Patients with ICC who underwent surgical treatment at Mengchao Hepatobiliary Hospital of Fujian Medical University between February 14, 2017, and March 12, 2021, were included in this study. A total of 50 fecal samples from healthy controls and 117 fecal samples along with 220 serum samples from ICC patients were collected. The cancer samples were categorized into four time periods: preoperative, postoperative day 7, 1–3 months postoperative, and postoperative recurrence. Inclusion criteria were as follows: (1) histologically confirmed intrahepatic cholangiocarcinoma and (2) availability of clinical information at the time of diagnosis. Patients lost to follow-up were excluded. All included patients met the specified inclusion and exclusion criteria. Samples were aseptically collected in the operating room and immediately stored at -80 °C for 30 minutes after collection.

### Sequencing and data processing

16S rRNA sequencing was employed for fecal microbiota analysis. Fresh fecal samples were collected from each subject using sterile spoons and immediately transferred into 3 mL of preservation solution to maintain sample integrity. To characterize the gut microbiota, DNA extraction was performed using the hexadecyltrimethylammonium bromide/sodium dodecyl sulfate (CTAB/SDS) method. The extracted DNA was quantified and diluted to a concentration of 1 mg/mL in sterile water to ensure consistency across samples. The V3-V4 regions of the 16S rRNA gene were amplified using barcoded primers 341F (5’-CCTACGGGNGGCWGCAG-3’) and 805R (5’-GACTACHVGGGTATCTAATCC-3’). PCR reactions were conducted using Phusion High-Fidelity PCR Master Mix (15 µL) from New England Biolabs. PCR products were then purified using a gel extraction kit (Qiagen, Hilden, Germany) following equimolar pooling. Sequencing libraries were prepared using an Illumina TruSeq DNA PCR-Free Sample Preparation Kit (Illumina, USA) and sequenced on the Illumina NovaSeq platform, generating 250-bp paired-end reads. Paired-end reads were merged using FLASH (v1.2.7) (19), and raw tags were quality-filtered using QIIME (v1.9.1) (20) to obtain clean, high-quality sequences. Chimera sequences were identified and removed using the UCHIME algorithm against the Silva database (21, 22). Sequences with ≥97% similarity were clustered into operational taxonomic units (OTUs) using UPARSE (v7.0.1001) (23). Taxonomic annotation was performed using the Mothur method and the Silva Database (24).

### Metagenomic sequencing

In this investigation, we utilized the PE150 sequencing mode on the NovaSeq 6000 platform for metagenomic sequencing. Raw sequencing reads were processed using Trimmomatic software to filter out adapter sequences and low-quality bases. Specifically, bases with quality scores less than five at the ends were trimmed, and sliding windows with an average quality score below fifteen were removed. Only sequences longer than 50 bp were retained after filtering. To eliminate host contamination, we used SOAP (25) to filter out sequences with more than 90% sequence alignment similarity to the host genome. After quality control and host sequence removal, the remaining sequences were considered effective data. For taxonomic profiling, we aligned the effective sequences against the Integrated Gene Catalog (IGC) of gut microbes (26) as a reference database. Sequences with greater than 90% similarity were retained for further analysis. The abundance of each gene in the IGC was calculated based on the number of matching sequences, normalized by gene length to obtain relative abundance values. Species annotation was performed using MetaPhlan 3.0 (27), following the recommended parameters outlined in the official documentation. Relative abundance information was calculated at the phylum, genus, and species levels using the built-in algorithms provided by the software.

### Metabolome sequencing

Each 100 μL serum sample was mixed with 400 μL of a methanol-acetonitrile (1:1 v/v) solution containing isotopically labeled internal standards. After vortexing for 30 seconds, samples were sonicated for 10 minutes in an ice-water bath and then incubated at -40 °C for 1 hour to precipitate proteins. Following centrifugation at 14,000 g for 15 minutes at 4 °C, the supernatants were transferred to separate glass vials for LC-MS/MS analysis. Aliquots of the supernatant from each sample were pooled to create the quality control (QC) sample.

### LC-MS/MS analysis

The LC-MS/MS analysis was performed using a UHPLC system (Vanquish, Thermo Fisher Scientific) coupled with a BEH Amide column (2.1 mm × 100 mm, 1.7 μm) and a Q Exactive HFX mass spectrometer (Orbitrap MS, Thermo). Mobile phase A consisted of 25 mM ammonium acetate and 25 mM ammonia hydroxide in water (pH = 9.75), while mobile phase B was acetonitrile. The injection volume was 2 μL, and the autosampler temperature was set to 4 °C. MS/MS spectra were collected using the QE HFX mass spectrometer in information-dependent acquisition mode, controlled by Xcalibur software (Thermo). The ESI source settings were as follows: sheath gas flow rate at 30 Arb, auxiliary gas flow rate at 25 Arb, capillary temperature at 350 °C, full MS resolution at 120,000, MS/MS resolution at 7500, spray voltage at 3.6 kV (positive mode) or -3.2 kV (negative mode), and collision energy at 10/30/60 in NCE mode.

### Data analysis

We used the R package “vegan” (28) to compute Shannon’s index and Invsimpson’s index for alpha diversity analysis. Beta diversity was assessed using nonmetric multidimensional scaling (NMDS) and principal coordinate analysis (PCoA), both based on the Canberra distance. Differences in microbial abundance among groups were visualized using volcano plots generated with the R packages “DESeq2” (29), “edgeR” (30), and “ggplot2” (31). The Kruskal-Wallis test was employed to analyze inter-group differences, with p < 0.05 indicating statistical significance. Principal component analysis (PCA) was conducted using the “FactoMineR” package (32).

KEGG enrichment analysis was performed using OmicShare tools, a free online platform for data analysis (https://www.omicshare.com/tools). Kaplan-Meier survival curves and Cox regression models were utilized to evaluate the impact of different time periods and key markers on patients’ overall survival (OS). Univariate and multivariate regression analyses identified relevant clinical indicators, important flora, and metabolites associated with ICC.

Correlation analysis results were visualized using the R packages “circlize” (33) and “ComplexHeatmap” (34) for correlation network visualization. Based on key microorganisms and metabolites, we trained a random forest model using the “randomForest” package (35) to predict cholangiocarcinoma recurrence and identify important features affecting recurrence.

Additionally, we applied the consensus clustering algorithm from the “CancerSubtypes” package (36), integrating 16S microbial and metabolomic data, to perform multiomics subtype identification. This approach aimed to discover potential ICC subtypes and elucidate the complex interactions and regulatory networks between microbes and host metabolism.

**Figure 9:**
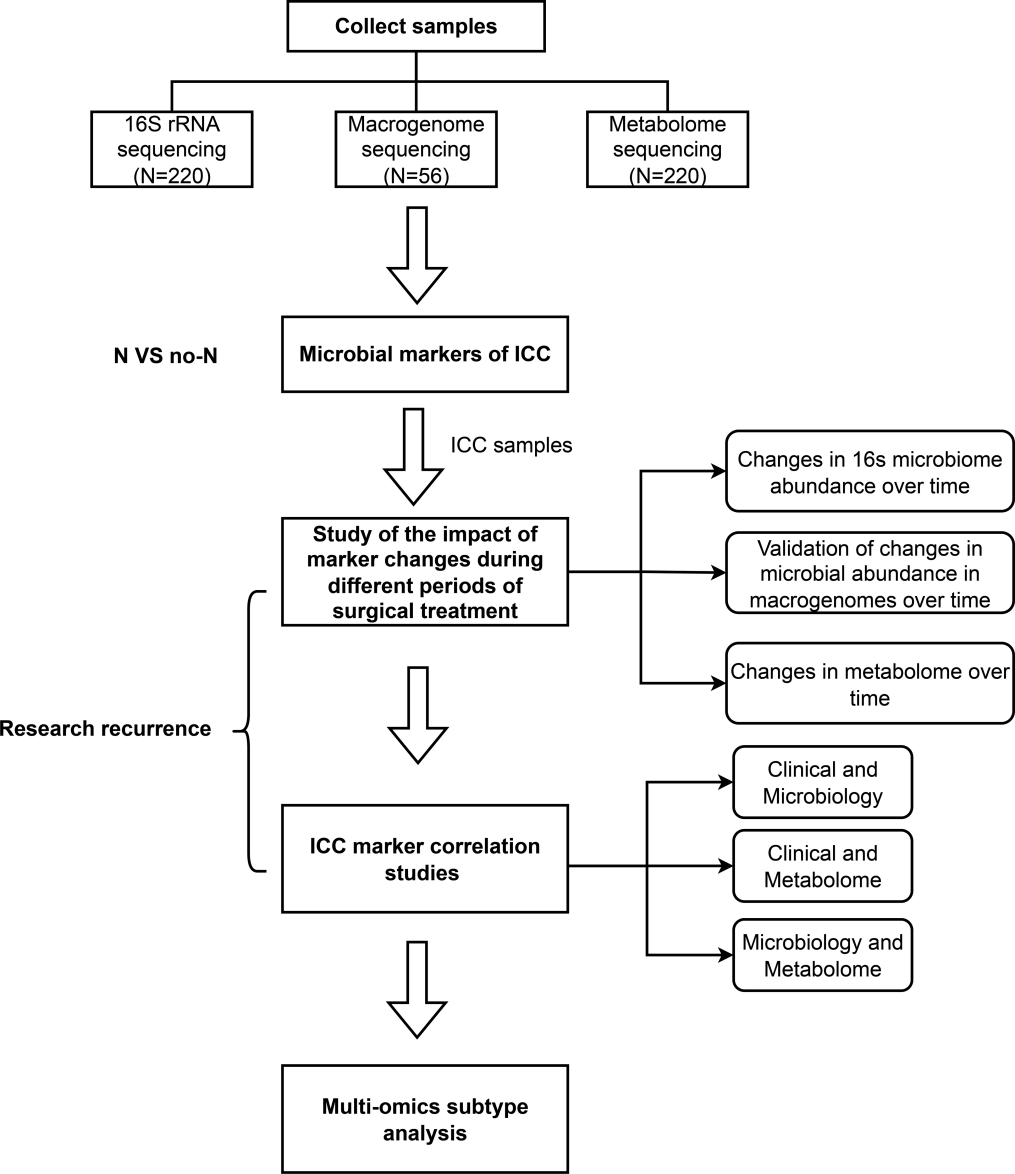
Overall flow chart. First, we investigated important microbial markers between ICC patients and normal controls using 16S sequencing. Next, we categorized the cancer samples according to different time periods and analyzed changes in microbial abundance and metabolites over time. To elucidate the interactions between clinical characteristics, the microbiome, and the metabolome, we conducted a two-by-two correlation analysis to identify key markers that significantly impacted ICC recurrence. Finally, we performed multiomics analysis using these key microbial and metabolite markers, leading to the identification of new ICC subtypes and an analysis of their associations with patient prognosis, as well as their relationships with specific microbes and metabolites.

Finally, we analyzed the biological differences and functional properties between different ICC subtypes and validated the key biomarkers that influence various stages of ICC. The overall analysis workflow is illustrated in **Figure 9**.

## Data Availability

The original contributions presented in the study are included in the article/**Supplementary Material**. Further inquiries can be directed to the corresponding authors.
