# Supplementary material for: Comprehensive identification of microbial and metabolomic factors impacting ICC recurrence
Source: Front Oncol. 2026 Feb 12;15:1703182. doi: 10.3389/fonc.2025.1703182 (PMC12935644; doi:10.3389/fonc.2025.1703182)
Supplement: Supplementary file 3 [file SupplementaryFile1.docx]

Supplementary Figure 1………………………………………………………………………….…2

Supplementary Table S1………………………………………………………………………….…2

Supplementary Table S2…………………………………………………………………....….……3

Supplementary Table S3……………………………………………………….………….………3

Supplementary Table S4……………………………………………………….……….………….3


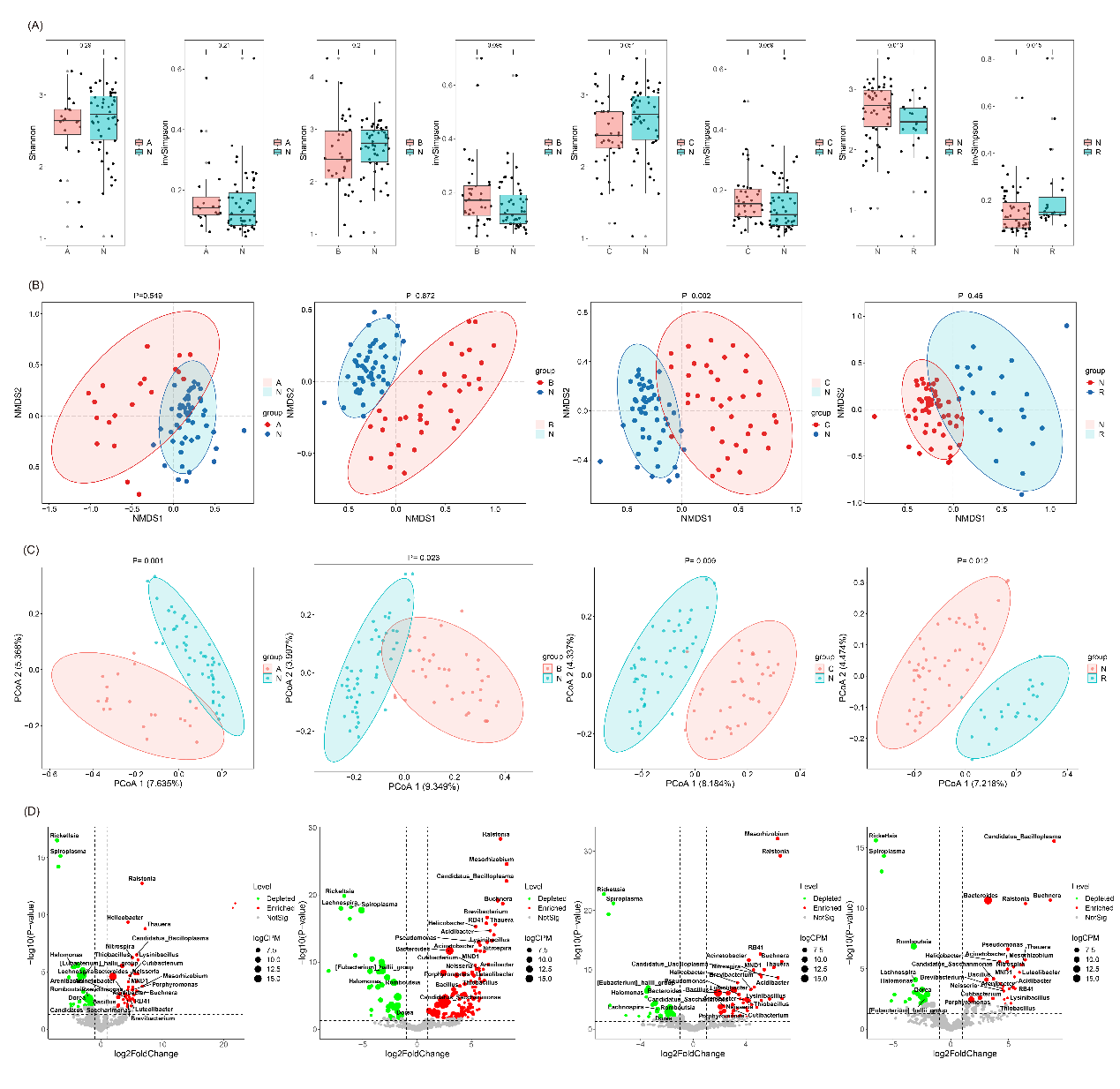


Supplementary Figure 1. In different periods in patients with bile duct carcinoma and normal group stool samples for 16 s rDNA sequencing.

(A) Box plots of Shannon index and anti-Simpson index (Invsimpson) between normal group and other groups (A, B, C, R). (B) Non-metric multidimensional scaling analysis (NMDS) based on Canberra distance between normal group and other groups (A, B, C, R). (C) Principal coordinate analysis (PCoA) based on Canberra distance between normal group and other groups (A, B, C, R). (D) the other group and normal group (A, B, C, R) in the relative abundance of the highest level 20 flora in the percentage of normal group and other groups of stacked bar chart. (E) the other group and normal group (A, B, C, R) level of bacterial abundance in volcanic figure. Truncation condition for (| log2 fold - change | > 1, p < 0.05), decreased significantly in the tissues of the bacteria display for the green; In the organization of bacteria showed obvious enrichment in red. Figure in the label of bacteria are four group were significantly raised or lowered significantly.

Table S1 Multivariate Cox regression analysis was performed with Veillonella and related clinical factors.

| **Characteristics** | **HR** | **95%CI** | **P** |
| --- | --- | --- | --- |
| **MONO** | 1 | 1-1.03 | 0.036 |
| **CA125** | 1 | 1-1.01 | 0.023 |
| **AFP** | 1 | 1-1 | 0.08 |
| **Veillonella** | 1.7 | 0.69-4.16 | 0.25 |

Table S2 Multivariate Cox regression analysis was performed with Abiotrophia and related clinical factors.

| **Characteristics** | **HR** | **95%CI** | **P** |
| --- | --- | --- | --- |
| **MONO** | 1 | 1-1.03 | 0.051 |
| **CA125** | 1 | 1-1.01 | 0.027 |
| **AFP** | 1 | 1-1.02 | 0.29 |
| **Abiotrophia** | 0.0052 | 1.3e-08-2130 | 0.42 |

Table S3 Multivariate Cox regression analysis was performed with Enterococcus and related clinical factors.

| **Characteristics** | **HR** | **95%CI** | **P** |
| --- | --- | --- | --- |
| **MONO** | 1 | 1-1.04 | 0.027 |
| **CA125** | 1 | 1-1.01 | 0.046 |
| **AFP** | 1 | 1-1 | 0.057 |
| **Enterococcus** | 0.64 | 0.25-1.66 | 0.36 |

Table S4 Multivariate Cox regression analysis was performed with Peptococcus and related clinical factors.

| **Characteristics** | **HR** | **95%CI** | **P** |
| --- | --- | --- | --- |
| **MONO** | 1 | 1-1.03 | 0.063 |
| **CA125** | 1 | 1-1.01 | 0.026 |
| **AFP** | 1 | 1-1 | 0.12 |
| **Peptococcus** | 2.2 | 0.87-5.76 | 0.095 |
